# Polymorphisms of Il-10 (-1082) and RANKL (-438) Genes and the Failure of Dental Implants

**DOI:** 10.1155/2017/3901368

**Published:** 2017-02-28

**Authors:** Rodrigo Ribeiro, Rayanne Melo, Pedro Tortamano Neto, André Vajgel, Paulo Roberto Eleutério Souza, Renata Cimões

**Affiliations:** ^1^Federal University of Campina Grande, Campina Grande, PB, Brazil; ^2^Federal University of Pernambuco, Recife, PE, Brazil; ^3^Department of Prosthodontics, University of São Paulo, São Paulo, SP, Brazil; ^4^Department of Genetics, Rural Federal University of Pernambuco, Recife, PE, Brazil; ^5^Department of Prosthodontics and Oral and Facial Surgery, Federal University of Pernambuco, Recife, PE, Brazil

## Abstract

*Background*. Genetic polymorphisms in certain cytokines and chemokines have been investigated to understand why some individuals display implant flaws despite having few risk factors at the time of implant.* Purpose*. To investigate the association of genetic polymorphisms in interleukin-* (IL-) 10* [-1082 region (A/G)] and* RANKL* [-438 region (A/G)] with the failure of dental implants.* Materials and Methods*. This study included 90 partially edentulous male and female patients who were rehabilitated with a total of 245 Straumann dental implants. An implant was considered a failure if any of the following occurred: mobility, persistent subjective complaint, recurrent peri-implant infection with suppuration, continuous radiolucency around the implant, probing depth ≥ 5 mm, and bleeding on probing. Buccal mucosal cells were collected for analysis of* RANKL438* and* IL-10*.* Results*. The implant success rate in this population was 34.4%. The mutant allele (G) in* RANKL* had an incidence of 52.3% and mutant allele (A) in* IL-10* was observed in 37.8%. No statistically significant difference was detected between the failure of the implant and the genotypes and allelic frequencies.* Conclusion*. No association was detected between the genetic polymorphisms of* RANKL* (-438) and* IL-10* (-1082) and the failure of dental implants in the population studied.

## 1. Introduction

Despite high dental implant survival rates, failures can occur, especially during initial healing and after the first year of loading. However, complications also arise during the maintenance phase of implants [1]. Several longitudinal studies have reported survival rates of about 85–95% for periods of 5–10 years [1–4].

Inflammation of peri-implant tissue occurs mainly due to the presence of bacteria and the consequent host immune response [5, 6]. This response can be regulated positively or negatively by a number of factors such as local and systemic diseases, medications, systemic hormones, local cytokines including interleukin (IL)-1, IL-6, and IL-10, growth factors, mediators of bone metabolism (RANK) [7, 8], and genetic polymorphisms [9]. Although pathogens are the known etiological agents in peri-implant inflammatory disease, subsequent progression and disease severity can be attributed to differences in the host response to pathogenic microorganisms, as observed in other infectious diseases [10].

Understanding the risk rate of peri-implantitis is important for treatment planning and prognosis, as prevention and treatment can be adjusted depending on the risk factors. However, routine diagnostic procedures, such as clinical and radiographic examination, often lack sufficient sensitivity to provide this information. In this regard, genetic biomarkers might be better suited for the estimation of this risk, as they are constant and can be measured before the onset of disease. Biochemical markers are more suitable for monitoring the onset, intensity, and activity of disease [11].

In the field of implantology, genetic polymorphisms in certain cytokines and chemokines have been investigated to understand why some individuals display implant flaws despite having few risk factors at the time of implant, good bone quality and quantity, no systemic disease or use of drugs, and appropriate surgical-prosthetic planning strategies [12]. Single nucleotide polymorphisms (SNPs) are the most common form of DNA variation in the human genome, and polymorphic alleles have been associated with increased susceptibility to complex human diseases [13–16].

Gene polymorphisms in an individual may modulate the severity and progression of inflammation by oral cytokine expression and modulation [17]. IL-10 is an anti-inflammatory cytokine that inhibits the production of proinflammatory cytokines [18], thereby disrupting the ability to induce the proliferation of B-lymphocytes and preventing the proliferation and activation of natural killer cells [19]. It is likely that IL-10 polymorphisms modulate the levels of protein and are associated with chronic periodontitis [20].

The RANK ligand (RANKL) receptor functions in activating the differentiation of monocytes into osteoclasts [21]. It is a key cytokine in the induction of osteoclastogenesis [22] and holds fundamental importance for the differentiation, activation, and survival of osteoclasts [23]. Regarding the relationship between polymorphisms of the* RANKL* gene and diseases related to increased bone resorption,* RANKL* has been suggested as one of the genetic determinants of inflammation in peri-implant tissues [24]. Thus, there are good arguments for a relationship between protein-coding genes of the innate immune response and inflammatory peri-implant disease. The relationship between genetic susceptibility and polymorphisms with respect to peri-implant complications is unclear [25]. Given the importance of innate immunity in maintaining the state of periodontal health and peri-implantation, the present study investigated whether there is an association between* RANKL* (-438) and* IL-10* (-1082) polymorphisms and the failure of dental implants.

## 2. Materials and Methods

This study was approved by the Ethics Committee in Research of the Federal University of Pernambuco-UFPE (number 01219612.7.0000.5208) (see Supplementary Material available online at https://doi.org/10.1155/2017/3901368). All volunteers consented to participate in the study by signing the informed consent form.

A retrospective clinical study was conducted in patients rehabilitated with dental implants during the previous 15 years at the Foundation for Scientific and Technological Development of Dentistry (Fundecto/USP), at the postgraduate implantology clinics. The data related to patients were collected through anamnesis, where it was found that the majority lost their teeth due to caries or periodontal disease. Regarding the implants, the following information was collected through clinical and radiographic examination: loading time, subjective complaints (pain, dysesthesia, foreign body sensation), mobility, and periodontal and radiographic examination. The loading period, in years, was categorized into three groups: Group 1: <1 year of loading; Group 2: ≥1 and <5 years of loading; and Group 3: ≥5 years of loading.

A total of 150 patients were called to participate in the study, and 126 attended the tests; 15 patients were excluded from the study; and 21 were lost due to the lack of appropriate genetic material. The final sample consisted of 90 individuals with a total of 245 implants.

The inclusion criteria were as follows: patients who were ≥18 years old, were partially edentulous, were rehabilitated exclusively with well-positioned Morse taper internal connection implants (Straumann, Waldenburg, Switzerland), reported good health, had negative medical history for chronic disease (diabetes mellitus, osteoporosis, and cardiovascular disease), and had no history of occlusal overload (bruxism and clenching). To avoid factors that could confuse the results of the study, we excluded smokers, those with periodontitis history, patients who received grafts in the surgical procedure for implant placement, those who used anticoagulant or chronic steroid medications, and patients who received radiation therapy or chemotherapy.

All patients were evaluated by the same researcher. An interview based on clinical history was performed using a standardised file developed for the study. After anamnesis, the clinical parameters of probing depth (PD), bleeding on probing (BOP), and gingival recession (GR) were recorded for all implants. To collect these data, a survey of four sites around each implant was performed using a PC15 periodontal probe (Trinity, São Paulo, Brazil). The measurements were rounded to the nearest millimetre.

After clinical examination, radiographs were taken at the periapical region of each implant using the parallelism technique conducted for radiological analysis. For bone level implants, bone loss was calculated on the mesial and distal sides of each implant, ranging from the junction of the prosthetic component to the level of the bone crest. For tissue level implants, bone loss was calculated from the most apical point of the transgingival portion of the implant to the bone crest level. This test was used to evaluate vertical bone loss and determine the presence of radiolucency around the implant. After the tests, professional biofilm control was performed, and oral hygiene instructions were given to the patients.

To determine implant failure, we applied the clinical and radiographic criteria described by Ong et al. (2008) [26], including the changes in bone level. Clinical evaluation was performed to determine whether the implant showed lack of mobility [27], absence of persistent subjective complaints (pain, foreign body sensation, and/or dysesthesia) [27], absence of recurrent peri-implant infection with suppuration [27], absence of continuous radiolucency around the implant [27], a probing depth no greater than 5 mm [28, 29], no bleeding on probing [28], and no annual vertical bone loss exceeding 0.2 mm (mesial or distal) after the first year of loading [30, 31].

Implant failure was recorded when either clinical or radiological criteria were detected.

Cell collection was performed by peeling of the buccal mucosa with the aid of a cytobrush (Kolplast, Ltd.). These cells were transferred to an Eppendorf tube containing 1 mL of saline and stored at −20°C for later DNA extraction and analysis.

DNA was extracted from the samples using the QIAamp DNA Kit (Qiagen; Hilden, Germany) according to the manufacturer's specifications. After extraction, the DNA was kept at −20°C until processing by polymerase chain reaction (PCR).

Amplification of the promoter region of* RANKL438* (rs2277438) was performed using the amplification-refractory mutation system- (ARMS-)PCR, as described by Kadkhodazadeh et al. (2013) [44].* RANKL-438* is located in the 5′ untranslated region (UTR) [32].

ARMS-PCR reactions were prepared using the reagent set GoTaq® Green Master Mix (a, b) (Madison, WI, USA) with the following reaction protocol: 5 *μ*L of water, 0.3 *μ*L of primer X (5′-GTTGGGGACATAAAGACTCTTGCA-3′), 0.3 *μ*L of common primer (5′-CTGCTATTTAATACAGTGTGACTTAAGAA-3′), 4.4 *μ*L of Master Mix, and 2 *μ*L of DNA sample, for a final volume of 12 *μ*L. The same protocol above was used for primer Y (5′-GGGGACATAAAGACTCTTGCG-3′). In all amplification reactions, amplification without a DNA sample was used as a negative control to check the possibility of contamination.

The thermocycler (Biocycler) was programmed as follows to amplify* RANKL438*: Phase 1 comprised the “hot start” (95°C for 5 min); phase 2 consisted of 35 cycles of three steps: DNA denaturation by heating (95°C for 1 min), annealing of the primer (53°C for 1 min), and extension (72°C for 1 min); Phase 3 encompassed the final extension (72°C for 7 min).

Next, 5 *μ*L of the PCR products was added to 4.0 *μ*L gel red fluorescent dye (Biotium, Brazil) and subjected to 2% agarose gel electrophoresis. Thereafter, electrophoresis runs were visualised under ultraviolet light and photographed for later analysis. The standard 100-bp molecular weight ladder (LGC Biotechnology, Brazil) was included in the electrophoresis run.

The polymorphism at 1082 of the* IL-10* gene was measured using the allele-specific PCR method. The primers used for detecting the SNP were (antisense) 5′-CAGTGCCAACTCAGAATTTGG-3′, primer G (sense) 5′-CTACTAAGGCTTCTTGAG-3′, and primer A (sense) 5′-ACTACTAAGGTCTCTTTGGAAA-3′. The PCR conditions were 95°C for 5 min, followed by 35 cycles at 95°C for 30 s, 62°C for 30 s, and 72°C for 30 s, followed by a final extension at 72°C for 5 min. The PCR product was 258 bp, which was visualised by agarose gel electrophoresis on 2% gel stained with red fluorescent dye (Biotium, Brazil).

The PCR reaction was run in a final volume of 10 *μ*L, with 5 *μ*L of Master Mix (Promega), 0.5 *μ*L of each sense primer (primers A and G), 0.5 *μ*L of antisense primer (primer concentrations were 10 mM), 2 *μ*L of water, and 2 *μ*L of DNA.

After collection, the data were analysed by IBM SPSS Statistics 20.0 trial version (IBM, Armonk, NY). The chi-squared test was used to compare the genotype frequencies (AA, AG, and GG) and alleles (A and G) for the* IL-10* gene (-1082 A/G) and to compare the genotype frequencies (AA, AG, and GG) and alleles (A and G) for the* RANKL* gene (-438) among the samples. Comparisons according to the criteria of mobility, subjective complaints, infection with suppuration, radiolucency, probing ≥ 5 mm, bleeding on probing, and annual vertical bone loss after implant were performed using Fisher's exact test. Data are expressed as absolute and relative values.

## 3. Results

This retrospective study included 90 patients, of whom 68.9% were female (*n* = 62). Patient age ranged between 21 and 80 years, with a mean of 54.5 years. The loading period of the implants ranged from 4 months to 15 years, and 50% of patients had had functional implants for more than 5 years. The failure rate in the population analysed was 34.4%, considering individuals.

The genotype analysis for* RANKL438* showed that the AG genotype was the most common (91.1%). The AA genotype was less frequent, with only two subjects (22.2%). The G allele (mutant) was present in 52.3% of the samples (Table 1).

In genotype analysis for the* IL-10* gene, the AG genotype was the most frequent, being present in 40 patients (44.4%); the AA genotype was present in only 18.9% of the samples. The A allele (mutant) was observed in 37.8% of cases (Table 1).

We found no significant association (*p* > 0.05) between the frequencies of genotypes in the* RANKL* gene region (-438 A/G) and implant failure. Taking into account the distribution of allele frequencies, it was also not possible to verify significant difference between A and G (*p* > 0.05) (Table 2).

We found no significant association (*p* > 0.05) between genotypic frequencies in the region of the* IL-10* gene (-1082 A/G) and implant failure. Regarding the distribution of allele frequencies, it was also not possible to verify significant differences between A and G (*p* > 0.05) (Table 2).

Considering the criteria referred to as major causes for the failure of the implants (radiolucency, probing ≥ 5 mm, bleeding on probing, and annual vertical bone loss), there was no significant association between the genotypes and the criteria.

## 4. Discussion

The host response against bacterial aggression seems to be a common denominator in peri-implant diseases, as well as in the understanding of genetic processes. It has become clear that the host response to bacterial invasion, inflammation, tissue destruction, and tissue repair are mediated by complex gene-gene and gene-environment interactions. There is evidence that flaws in dental implants in certain subsets of subjects [33, 34] may indicate that specific characteristics of the host, such as genetic factors, play a role in the process of osseointegration [35].

The presence of SNPs in immune cytokine genes is considered an important factor for genetic diversity of the host, as it influences the ability of production of the cytokines, changing the transcription and cytokine gene expression [36]. These polymorphisms can also act as a factor for predisposition to diseases according to the expression of interleukins [37]. Thus, research on genetic polymorphisms might reveal determining factors for patient prognosis.

This study compared the frequency of polymorphisms in* IL-10* (-1082) and* RANKL* (-438) and investigated a possible association with failures in dental implants. These polymorphisms could be involved in the deregulation of cytokine expression, directly affecting the balance between anti- and proinflammatory proteins, which could affect the success or failure of the implants evaluated in this study. Teixeira et al. (2014) [38] also evaluated the association between failures of dental implants and polymorphism in RANKL (-438) and did not observe a significant relationship with any genotypes in this sample. Sites with peri-implant inflammation have increased gum bleeding and crevicular fluid, which form an exudate [39]. It has been proposed that the levels of inflammatory mediators identify active peri-implantitis, providing a promising diagnostic and preventive tool for the disease [40].

Similarities in the frequency distribution of alleles and genotypes of the groups suggest a possible relationship between immunogenetic implant failure and chronic periodontitis [41].

Thomas et al. (2013) [42] used mononuclear cells (lymphocytes, monocytes, and plasma cells) to compare the* IL-10* expression in patients without implants and symptom-free patients with implants. None of the 14 individuals without implants group showed* IL-10* expression, whereas five of the six individuals with implants showed IL-10 expression.

Gurol et al. (2011) [41] evaluated 39 patients with implants and found no statistical relationship between polymorphism of* IL-10* (-1082) and implant failure. Even with a considerable increase in sample size, our study confirms these findings. These results also agree with another study [43] that found no association between polymorphism of the IL-10 gene and chronic periodontitis.

In relation to allelic frequency, Gurol et al. (2011) [41] noted that the A allele at position -1082 was more prevalent in healthy subjects (63%) than in those with implant failure (50%), chronic periodontitis (47%), and healthy implants (48%), a result similar to that found in this study. Moreover, the same authors reported that the frequencies of alleles at -1082 were relatively equally distributed (50%) in the patients with chronic periodontitis, healthy implants, and implant failure. It was also observed that the AG genotype predominated in patients with chronic periodontitis. Our study did not find an association between allelic frequency and the failure of implants, despite the high incidence of the mutant allele found for IL-10 (-1082).

RANKL is a key factor for osteoclast differentiation and activation [44] and is positively correlated with probing depth and the level of clinical attachment. Moreover, higher levels are reported in patients with periodontitis than in patients with good gingival health [36, 45, 46]. Thus, the* RANKL* gene may be one of the genetic determinants of periodontitis and peri-implantitis, based on other diseases attributed to osteoclastic bone resorption [24].

In this study, we found no significant association between the genotypes of* RANKL* (-438) and the failure of implants, despite the high incidence of the mutant allele. Corroborating these data, Kadkhodazadeh et al. (2013) [44] observed that the expression of* RANKL* genotypes (-438) was not significantly different between patients with healthy periodontium and those with chronic periodontitis and peri-implantitis; the allelic frequency did not differ among these three groups.

In their systematic review, Duarte et al. (2016) [47] investigated whether the levels of crevicular fluid cytokines could be used to distinguish between healthy implants and peri-implantitis. The authors noted that most studies found no significant differences in the levels of anti-inflammatory cytokines (such as IL-10) and RANKL between healthy implants and implants with peri-implantitis. They also showed that the levels of anti-inflammatory cytokines, cytokines related to osteoclastogenesis, and chemokines were severely limited as potential predictors of peri-implantitis.

Future diagnostic and therapeutic methods may be based on genetic characteristics of the individual, which may be either protective or destructive, according to the identification of polymorphic regions. Such methods might provide information supporting the identification of high-risk patients for implant placement, development of surgical protocol, and efforts toward preservation, in addition to other advantages [12]. However, our results suggest that* RANKL *(-438) and* IL-10 *(-1082) should not be considered for this purpose. In this study population, the evaluated polymorphisms were not predictive of dental implant failure.

## Supplementary Material

This number is an endorsement for the researcher, demonstrating that he presented his project for ethical approval.

## Figures and Tables

**(a):** 

RANKL (-438)		*N*	%
Genotypes	AA	2	2.2
	AG	82	91.1
	GG	6	6.7

Alleles	A	86	4.7
	G	94	52.3

**(b):** 

IL-10		*N*	%
Genotypes	GG	33	36.7
	AG	40	44.4
	AA	17	18.9

Alleles	A	86	41.1
	G	94	58.9

**(a):** 

RANKL (-438)		Failure		Total	*p* value^1^
		Yes	No		
Genotypes	AA	0 (0%)	2 (100%)	2 (100%)	0.416
	AG	28 (34.1%)	54 (65.9%)	82 (100%)	
	GG	1 (16.7%)	5 (83.3%)	6 (100%)	

Alleles	A	28 (32.6%)	58 (67.4%)	86 (100%)	0.927
	G	30 (31.9%)	64 (68.1%)	94 (100%)	

**(b):** 

IL-10 (-1082)		Failure		Total	*p* value^1^
		Yes	No		
Genotypes	AA	6 (35.3%)	11 (64.7%)	17 (100%)	0.222
	AG	16 (40%)	24 (60.0%)	40 (100%)	
	GG	7 (21.2%)	26 (78.8%)	33 (100%)	

Alleles	A	28 (37.8%)	46 (62.2%)	74 (100%)	0.178
	G	30 (28.3%)	76 (71.7%)	106 (100%)	

^1^Pearson chi-square test.

## References

[1] Esposito M., Hirsch J.-M., Lekholm U., Thomsen P. (1998). Biological factors contributing to failures of osseointegrated oral implants: (II). Etiopathogenesis. *European Journal of Oral Sciences*.

[2] Berglundh T., Persson L., Klinge B. (2002). A systematic review of the incidence of biological and technical complications in implant dentistry reported in prospective longitudinal studies of at least 5 years. *Journal of Clinical Periodontology*.

[3] Roos-Jansåker A. M., Lindahl C., Renvert H., Renvert S. (2006). Nine- to fourteen-year follow-up of implant treatment. Part I: implant loss and associations to various factors. *Journal of Clinical Periodontology*.

[4] Holm-Pedersen P., Lang N. P., Müller F. (2007). What are the longevities of teeth and oral implants?. *Clinical Oral Implants Research*.

[5] Garg A. (2010). Peri-implant disease: the basics. *Dental implantology update*.

[6] Lang N. P., Berglundh T. (2011). Periimplant diseases: where are we now?—Consensus of the Seventh European Workshop on periodontology. *Journal of Clinical Periodontology*.

[7] Soedarsono N., Rabello D., Kamei H. (2006). Evaluation of RANK/RANKL/OPG gene polymorphisms in aggressive periodontitis. *Journal of Periodontal Research*.

[8] Baioni C. S., De Souza C. M., Ribeiro Braosi A. P. (2008). Analysis of the association of polymorphism in the osteoprotegerin gene with susceptibility to chronic kidney disease and periodontitis. *Journal of Periodontal Research*.

[9] Kornman K. S., Crane A., Wang H. Y. (1997). The interleukin-1 genotype as a severity factor in adult periodontal disease. *Journal of Clinical Periodontology*.

[10] Medzhitov R. (2007). Recognition of microorganisms and activation of the immune response. *Nature*.

[11] Rakic M., Petkovic-Curcin A., Struillou X., Matic S., Stamatovic N., Vojvodic D. (2015). CD14 and TNF*α* single nucleotide polymorphisms are candidates for genetic biomarkers of peri-implantitis. *Clinical Oral Investigations*.

[12] Gonçalvez R, Coelho R., Barboza E. P., Granjeiro J. M., Casado P. (2011). A característica genética influência na sobrevida do implante dentário?. *Bazilian Journal of Periodontology*.

[13] Asensi V., Alvarez V., Valle E. (2003). IL-1*α* (-889) promoter polymorphism is a risk factor for osteomyelitis. *American Journal of Medical Genetics*.

[14] Trevilatto P. C., Scarel-Caminaga R. M., de Brito R. B., de Souza A. P., Line S. R. (2003). Polymorphism at position-174 of IL-6 gene is associated with susceptibity to chronic periodontitis in a Caucasian Brazilian population. *Journal of Clinical Periodontology*.

[15] Gruica B., Wang H.-Y., Lang N. P., Buser D. (2004). Impact of IL-1 genotype and smoking status on the prognosis of osseointegrated implants. *Clinical Oral Implants Research*.

[16] Riemenschneider M., Konta L., Friedrich P. (2006). A functional polymorphism within plasminogen activator urokinase (PLAU) is associated with Alzheimer's disease. *Human Molecular Genetics*.

[17] Lachmann S., Kimmerle-Müller E., Axmann D., Scheideler L., Weber H., Haas R. (2007). Associations between peri-implant crevicular fluid volume, concentrations of crevicular inflammatory mediators, and composite IL-1A -889 and IL-1B + 3954 genotype: a cross-sectional study on implant recall patients with and without clinical signs of peri-implantitis. *Clinical Oral Implants Research*.

[18] Reinhardt R. A., Masada M. P., Johnson G. K., DuBois L. M., Seymour G. J., Allison A. C. (1993). IL-1 in gingival crevicular fluid following closed root planing and papillary flap debridement. *Journal of Clinical Periodontology*.

[19] Moore K. W., De Waal Malefyt R., Coffman R. L., O'Garra A. (2001). Interleukin-10 and the interleukin-10 receptor. *Annual Review of Immunology*.

[20] Scarel-Caminaga R. M., Trevilatto P. C., Souza A. P., Brito R. B., Camargo L. E. A., Line S. R. P. (2004). Interleukin 10 gene promoter polymorphisms are associated with chronic periodontitis. *Journal of Clinical Periodontology*.

[21] Xiong D.-H., Shen H., Zhao L.-J. (2006). Robust and comprehensive analysis of 20 osteoporosis candidate genes by very high-density single-nucleotide polymorphism screen among 405 white nuclear families identified significant association and gene-gene interaction. *Journal of Bone and Mineral Research*.

[22] Suda T., Takahashi N., Udagawa N., Jimi E., Gillespie M. T., Martin T. J. (1999). Modulation of osteoclast differentiation and function by the new members of the tumor necrosis factor receptor and ligand families. *Endocrine Reviews*.

[23] Shalhoub V., Faust J., Boyle W. J. (1999). Osteoprotegerin and osteoprotegerin ligand effects on osteoclast formation from human peripheral blood mononuclear cell precursors. *Journal of Cellular Biochemistry*.

[24] Park O.-J., Shin S.-Y., Choi Y. (2008). The association of osteoprotegerin gene polymorphisms with periodontitis. *Oral Diseases*.

[25] Loss B. G., Van Der Velden U., Laine M. L., Lindhe J., Lang N., Karring T. (2008). Suceptibility. *Clinical Periodontology and Implant Dentistry*.

[26] Ong C. T. T., Ivanovski S., Needleman I. G. (2008). Systematic review of implant outcomes in treated periodontitis subjects. *Journal of Clinical Periodontology*.

[27] Buser D., Weber H. P., Lang N. P. (1990). Tissue integration of non-submerged implants. 1-year results of a prospective study with 100 ITI hollow-cylinder and hollow-screw implants. *Clinical Oral Implants Research*.

[28] Mombelli A., Lang N. P. (1994). Clinical parameters for the evaluation of dental implants. *Periodontology 2000*.

[29] Brägger U., Aeschlimann S., Bürgin W., Hämmerle C. H. F., Lang N. P. (2001). Biological and technical complications and failures with fixed partial dentures (FPD) on implants and teeth after four to five years of function. *Clinical Oral Implants Research*.

[30] Albrektsson T., Zarb G., Worthington P., Eriksson A. R. (1986). The long-term efficacy of currently used dental implants: a review and proposed criteria of success. *International Journal of Oral & Maxillofacial Implants*.

[31] Albrektsson T., Isidor F., Lang N. P., Karring T. (1994). Concensus report of session IV. *Proceedings of the 1st European Workshop on Periodontology*.

[32] Hsu Y.-H., Niu T., Terwedow H. A. (2006). Variation in genes involved in the RANKL/RANK/OPG bone remodeling pathway are associated with bone mineral density at different skeletal sites in men. *Human Genetics*.

[33] Hutton J. E., Heath M. R., Chai J. Y. (1995). Factors related to success and failure rates at 3-year follow-up in a multicenter study of overdentures supported by Brånemark implants. *The International journal of oral & maxillofacial implants*.

[34] Ekfeldt A., Christiansson U., Eriksson T. (2001). A retrospective analysis of factors associated with multiple implant failures in maxillae. *Clinical Oral Implants Research*.

[35] Santos M. C. L. G., Campos M. I. G., Line S. R. P. (2002). Early dental implant failure: a review of the literature. *Brazilian Journal Oral Sciences*.

[36] Chen B., Zeng Z., Xu L. (2011). IL23R +2199A/C polymorphism is associated with decreased risk of certain subtypes of gastric cancer in Chinese: a case-control study. *Cancer Epidemiology*.

[37] Poli F., Nocco A., Berra S. (2002). Allele frequencies of polymorphisms of TNFA, IL-6, IL-10 and IFNG in an Italian Caucasian population. *European Journal of Immunogenetics*.

[38] Teixeira C., Matheus T., Souza P., Donos N., Cimões R. (2014). Genetic polymorphism and failure of dental implants: a pilot study. *Dental Press Implantology*.

[39] Murata M., Tatsumi J.-I., Kato Y. (2002). Osteocalcin, deoxypyridinoline and interleukin-1*β* in peri-implant crevicular fluid of patients with peri-implantitis. *Clinical Oral Implants Research*.

[40] Petković A., Matić S., Stamatović N. (2010). Proinflammatory cytokines (IL-1*β* and TNF-*α*) and chemokines (IL-8 and MIP-1*α*) as markers of peri-implant tissue condition. *International Journal of Oral and Maxillofacial Surgery*.

[41] Gurol C., Kazazoglu E., Dabakoglu B., Korachi M. (2011). A comparative study of the role of cytokine polymorphisms interleukin-10 and tumor necrosis factor alpha in susceptibility to implant failure and chronic periodontitis. *The International Journal of Oral & Maxillofacial Implants*.

[42] Thomas P., Iglhaut G., Wollenberg A., Cadosch D., Summer B. (2013). Allergy or tolerance: reduced inflammatory cytokine response and concomitant il-10 production of lymphocytes and monocytes in symptom-free titanium dental implant patients. *BioMed Research International*.

[43] Kinane D. F., Hodge P. J., Eskdale J., Ellis R., Gallagher G. (1999). Analysis of genetic polymorphisms at the interleukin-10 and tumour necrosis factor loci in early-onset periodontitis. *Journal of Periodontal Research*.

[44] Kadkhodazadeh M., Ebadian A. R., Gholami G. A., Khosravi A., Tabari Z. A. (2013). Analysis of RANKL gene polymorphism (rs9533156 and rs2277438) in Iranian patients with chronic periodontitis and periimplantitis. *Archives of Oral Biology*.

[45] Mogi M., Otogoto J., Ota N., Togari A. (2004). Differential expression of RANKL and osteoprotegerin in gingival crevicular fluid of patients with periodontitis. *Journal of Dental Research*.

[46] Bostanci N., Ilgenli T., Emingil G. (2007). Gingival crevicular fluid levels of RANKL and OPG in periodontal diseases: implications of their relative ratio. *Journal of Clinical Periodontology*.

[47] Duarte P. M., Serrão C. R., Miranda T. S. (2016). Could cytokine levels in the peri-implant crevicular fluid be used to distinguish between healthy implants and implants with peri-implantitis? A systematic review. *Journal of Periodontal Research*.

