# Supplementary figures and images for: Polymorphisms of Il-10 (-1082) and RANKL (-438) Genes and the Failure of Dental Implants
Source: Int J Dent. 2017 Feb 28;2017:3901368. doi: 10.1155/2017/3901368 (PMC5350407; doi:10.1155/2017/3901368)

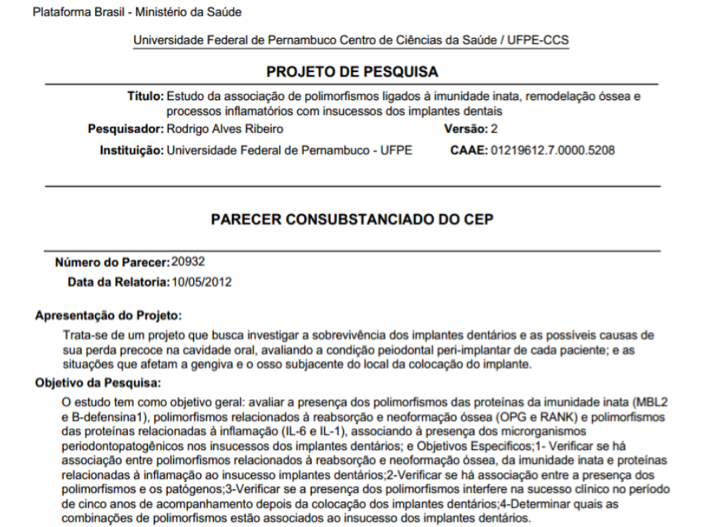


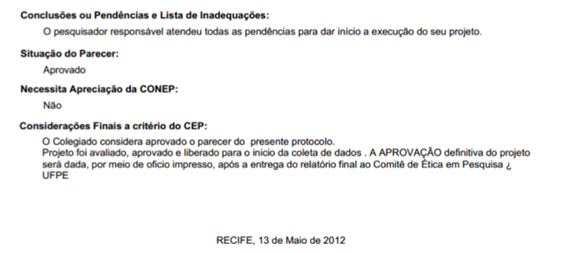

Supplement: Supplementary file 1 — This number is an endorsement for the researcher, demonstrating that he presented his project for ethical approval. [file 3901368.f1.doc]
